# Poliomyelitis

**Published:** 1917-09

**Authors:** 


**Poliomyelitis.** Horace Greeley, L. I. Med. Jour., April, collects the following notes as to occurrence in breast-fed infants: N. Y. 1907, 121 breast fed among 283 cases under 2 years; Ia. 1910, 2 among 47 considered; Cincinnati 1911, 15 among 83 considered; Buffalo 1912, 12 among 40 considered. Several negative and vague reports are also included. We have already mentioned his statistics as to the correspondence of incidence with high summer temperature.

